# No evidence of delay in colorectal cancer diagnosis during the COVID-19 pandemic in Gwangju and Jeonnam, Korea

**DOI:** 10.4178/epih.e2022092

**Published:** 2022-10-17

**Authors:** Hye-Yeon Kim, Min-Gyeong Kim, Mi-Ran Kang, Jeong-Ho Yang, Min-Ho Shin, Sun-Seog Kweon

**Affiliations:** 1 Department of Preventive Medicine, Chonnam National University Medical School, Hwasun, Korea; 2 Gwangju/Jeonnam Cancer Registry, Chonnam National University Hwasun Hospital, Hwasun, Korea; 3 Gwangju-Jeonnam Regional Cancer Center, Chonnam National University Hwasun Hospital, Hwasun, Korea

**Keywords:** COVID-19, Colorectal neoplasm, Neoplasm staging

## Abstract

**OBJECTIVES:**

We evaluated whether the coronavirus disease 2019 (COVID-19) pandemic caused delays in the diagnosis and treatment of colorectal cancer (CRC) in Korea, where there have been no regional or hospital lockdowns during the pandemic period.

**METHODS:**

Data on CRC patients (n=1,445) diagnosed in Gwangju Metropolitan City and Jeonnam Province between January 2019 and December 2021 were assessed. The stage at the time of CRC diagnosis, route to diagnosis, time to initial cancer treatment, and length of hospital admission were compared before and during the COVID-19 pandemic. Logistic regression was also performed to identify factors associated with the risk for diagnosis in an advanced stage.

**RESULTS:**

No negative effects indicating a higher CRC stage at diagnosis or delayed treatment during the pandemic were observed. Instead, the risk for an advanced stage at diagnosis (TNM stage III/IV) decreased in CRC patients diagnosed during the pandemic (odds ratio, 0.768; 95% confidence interval, 0.647 to 0.911). No significant differences in the interval from diagnosis to operation or chemotherapy were observed.

**CONCLUSIONS:**

No negative effects on CRC diagnosis and treatment were found until the end of 2021, which may be related to the small magnitude of the COVID-19 epidemic, the absence of a lockdown policy in Korea, and the rebound in the number of diagnostic colonoscopy procedures in 2021.

## GRAPHICAL ABSTRACT

[Fig f2-epih-44-e2022092]

## INTRODUCTION

As the coronavirus disease 2019 (COVID-19) pandemic has continued globally since December 2019, it has had extensive negative effects on public health, social welfare, and the economy. No one has remained unaffected, directly or indirectly, by this novel virus and its variants. As the pandemic period has been prolonged and the affected area has been widened, concerns about collateral damage to non-COVID-19 care, in addition to the direct impacts of COVID-19 itself, have grown. Governmental policies such as social distancing and staying indoors, lockdown of the health care delivery system, and fear of patients contracting COVID-19 in medical facilities have curtailed the use of essential medical care services [1]. In particular, studies are being conducted to identify the impacts of the COVID-19 pandemic on preventive services such as cancer screening [2]. Periodic cancer screening or the clinical diagnosis of suspected cancer symptoms could be delayed during the pandemic. As a result, the proportion of cancers diagnosed at an advanced stage might increase during and after the pandemic.

Although all types of cancer screening activities may be suspended or considerably slowed down during a pandemic, colorectal cancer (CRC) screening may be affected more than others since it involves multistep tests or invasive tests such as colonoscopy [3,4]. Several papers have been published about COVID-19-related missed or delayed CRC screenings and shifts in stage and survival since the COVID-19 pandemic began. Since the pandemic started, the fecal immunochemical testing (FIT) screening uptake rate has decreased in Taiwan [5], the interval between positive FIT and colonoscopy has become longer in Spain [6], and colonoscopy procedures have decreased by 55% in the Netherlands [7]. In April 2020, colonoscopy, colon cancer, and the CRC screening rate in the United States declined by 55.0%, 15.4%, and 37.7%, respectively [8]. In Korea, a nationwide survey reported that the colorectal cancer screening rate in 2020 decreased by 23% compared to 2019 [9].

Delaying colonoscopy more than 6–9 months after positive fecal testing is associated with higher risks of CRC and advanced-stage CRC [10,11]. An Italian modeling study estimated that screening delays beyond 4–6 months would increase the risk for advanced CRC and that mortality would also increase if screening delays lasted beyond 12 months during the COVID-19 pandemic period [12]. In England, a 15.3–16.6% increase in CRC deaths is expected as a result of diagnostic delays due to the COVID-19 pandemic [13]. However, the impact of the pandemic on CRC screening differs by country [14], which may be due to the stringency of lockdown policies by country, among other factors. In Korea, it was reported that there was no CRC stage upshifting during the early days of the COVID-19 pandemic [15].

Indeed, the magnitude of the pandemic has been smaller in Korea than in most Western countries. As of July 4, 2022, the numbers of cumulative COVID-19 cases and deaths per million population in Korea were 358,911.04 cases and 479.02 deaths, compared to 69,845.69 cases and 805.1 deaths per million population globally [16]. The Korean government officially first applied a social distancing policy in March 2020 to control the pandemic. However, there have been no hospital closures or regional or national lockdowns in Korea. Therefore, this study investigated whether delays in the diagnosis and treatment of CRC have occurred as an impact of the COVID-19 pandemic in Korea.

## MATERIALS AND METHODS

### Data collection

Data on CRC patients diagnosed from January 2019 to December 2021 were collected from the database of the Gwangju and Jeonnam cancer registries and medical records of Chonnam National University Hwasun Hospital (CNUHH). Gwangju is a metropolitan city with a population of 1.45 million, and Jeonnam is a predominantly rural area with a population of 1.85 million at the end of 2021. During the 3 years (2019–2021), a total of 2,379 CRC patients (1,457 males and 922 females) were newly diagnosed and received cancer treatment at the CNUHH (C18-C20 based on the 10th edition of the International Classification of Diseases). Among them, the number of CRC cases diagnosed in 2019 (n=798) accounted for 72.7% of total CRC incident cases in Gwangju and Jeonnam Province in 2019 (n=1,097), according to the annual report of community-based cancer registries [17]. The total number of CRC incident cases in Gwangju and Jeonnam Province in 2020 and 2021 has not been confirmed. Demographic data, including age, sex, address (rural or urban), family history of CRC, smoking and alcohol drinking status, the presence of diabetes mellitus (DM), body mass index (BMI), and information about the route of CRC diagnosis were collected by reviewing medical records. Addresses were classified into 2 groups: urban (Gwangju and 5 cities in Jeonnam) and rural (the remaining 17 administrative areas in Jeonnam, referred to as counties [*gun*]). The routes to CRC diagnosis were divided into 3 categories: diagnosis after symptom onset, via routine CRC screening, and via follow-up examinations for related diseases or other types of cancer. Clinical data, including TNM stage at cancer diagnosis, cancer treatment method, dates of CRC diagnosis and admission to hospital, and cancer treatment start date were identified. TNM stages were classified based on the eighth edition of the American Joint Committee for Cancer. The interval between the date of the first CRC confirmation by biopsy, regardless of hospital, and the date of initiation of cancer treatment in CNUHH was measured for surgery (diagnosis-operation interval), chemotherapy (diagnosis-chemotherapy interval), and radiation therapy (diagnosis-radiotherapy interval) to treat CRC. In addition, data were divided into pre-pandemic and pandemic periods according to whether the first date of CRC diagnosis took place before or after the launch of the national social distancing policy in Korea (February 29, 2020). Delayed treatment was defined as a diagnosis-treatment interval exceeding the median value of each interval during the pre-pandemic period (27, 50, and 31 days for delayed surgery, delayed chemotherapy, and radiotherapy, respectively). The CRC screening rate of the National Cancer Screening Program (NCSP), which is a free-of-charge organized cancer screening program targeting individuals with economic status in the lower half of the population, was obtained from the official dataset of the National Health Insurance Service for the same period (2018–2021). The number of diagnostic colonoscopies performed at all medical facilities in Gwangju and Jeonnam during 2018–2021 was measured using the database of the Korean Health Insurance Review and Assessment Services (claim code E7660).

### Statistical analysis

To evaluate whether there was a delay in the diagnosis or treatment of CRC due to the implementation of social distancing after the outbreak of the COVID-19 pandemic, demographic and behavioral characteristics, the route to diagnosis, the stage at diagnosis, present illness or past history, the length of admission, and the time interval from diagnosis to treatment were compared between CRC patients diagnosed during the pre-pandemic and pandemic periods. Continuous variables are presented as median values with interquartile ranges, and categorical variables are reported as frequencies with percentages. The Mann-Whitney U test and the chi-square test were applied for nonparametric data and categorical data, as appropriate. A 2-sided p-value of less than 0.05 was considered statistically significant. Odds ratios (ORs) and 95% confidence intervals (CIs) were also calculated to compare the risk for advanced stage (TNM stage III or IV) CRC at diagnosis and for delayed treatment, including delayed surgery, delayed chemotherapy, and delayed radiotherapy, using logistic regression. Sensitivity analyses were conducted excluding CRC cases diagnosed in the first half of 2020, since those cases might not yet have reflected the impact of social distancing or the pandemic. Additional logistic regression in which an advanced stage was defined as TNM stage IV was also performed to confirm the main findings.

### Ethics statement

The study protocol conformed to the ethical guidelines of the 1975 Declaration of Helsinki and was approved by the Institutional Review Board of CNUHH (No. CNUHH-2021-061).

## RESULTS

In total, 2,379 patients were newly diagnosed with CRC during the study period; 934 cases were diagnosed before the pandemic (pre-pandemic period) and 1,445 cases during the pandemic. In the cases diagnosed during the pandemic period, the proportions of early-stage cases (TNM stage I or II) and smokers were higher, and the length of admission was shorter than in those diagnosed in the pre-pandemic period. However, the proportion of CRC patients receiving chemotherapy was higher in the pandemic group. No significant differences were found in the distributions of age, sex, residency, route to diagnosis, anatomical sites, surgery, radiation therapy, BMI, alcohol drinking, family history of CRC, DM, and treatment time intervals (Table 1).

Findings from multivariate logistic regression analyzing the factors associated with the risk for advanced stage at CRC diagnosis are presented in Table 2. Age under 65 years and a diagnosis due to clinical symptoms were associated with a higher for advanced stage (TNM stage III/IV) at diagnosis, with ORs of 1.240 (95% CI, 1.042 to 1.476) and 2.496 (95% CI, 2.093 to 2.978). The risk for advanced stage was lower in CRC cases diagnosed during the pandemic period than in those diagnosed in the pre-pandemic period (OR, 0.768; 95% CI, 0.647 to 0.911), and higher in CRC cases diagnosed in patients who drank alcohol (OR, 1.219; 95% CI, 1.007 to 1.476). A non-obese BMI (<25 kg/m^2^, with ≥25 kg/m^2^ as the reference group) was also inversely associated with the risk for an advanced stage at diagnosis (OR, 1.253; 95% CI, 1.043 to 1.505). No association was found with sex, family history of CRC, residential area, DM, or smoking. Most of the findings did not change in sensitivity analyses (data not shown). However, in an additional analysis for the risk of metastasis, no significant difference between the CRC cases diagnosed in the pre-pandemic period and those diagnosed in the pandemic period was observed (OR, 0.926; 95% CI, 0.742 to 1.156) (Table 2).

The risks of delayed surgery, delayed chemotherapy, and delayed radiotherapy were not different between the pre-pandemic and pandemic periods. The risk of delayed treatment was lower in patients who received a symptomatic diagnosis than in those who were diagnosed via screening. The risk of delayed chemotherapy was lower in TNM stage III/IV cases, and the risk of delayed radiotherapy was lower in patients aged <65 years (Table 3).

**Figure 1:**
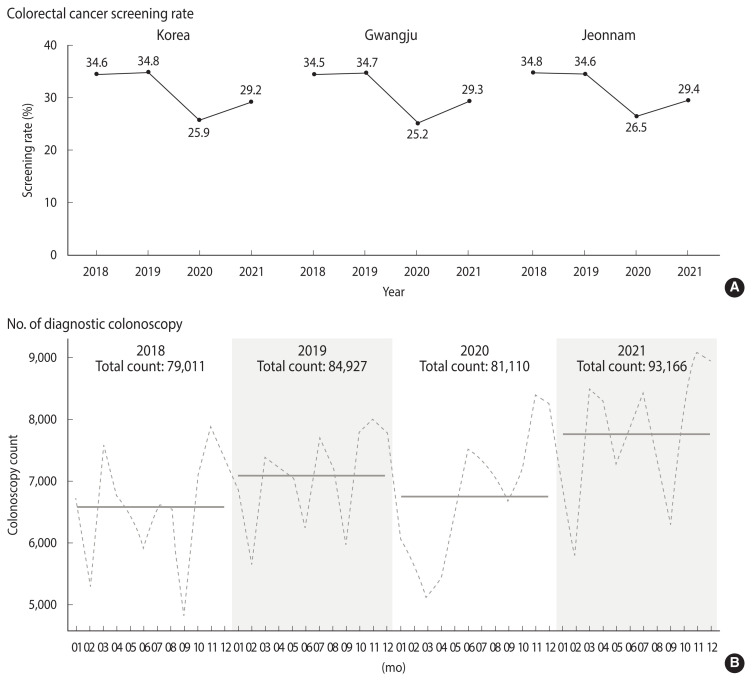
The changes of colon cancer screening rate and number of diagnostic colonoscopy during the pre-pandemic and pandemic period. (A) Colon cancer screening rates of National Cancer Screening Program in Korea, Gwangju, and Jeonnam, (B) total count of diagnostic colonoscopy performed in Gwangju and Jeonnam. Dotted line: monthly count of colonoscopy, solid line: annual average count of colonoscopy.

The national CRC screening rate in the NCSP in 2020 and 2021 decreased compared to the previous 2 years, both in Korea as a whole and in the study areas (Gwangju Metropolitan City and Jeonnam Province). The number of colonoscopies performed at all medical facilities in the study areas decreased in 2020, but rebounded to more than the previous levels in 2021 (Figure 1).

## DISCUSSION

We evaluated whether there were any delays in the diagnosis of CRC or initial cancer treatment in Korea during the COVID-19 pandemic in 2020 and 2021. No difference was observed in the basic characteristics of CRC patients between the pre-pandemic and pandemic periods; instead, decreases in the risk for advanced stage and the length of hospital admission stay for cancer treatment were observed. These findings are not consistent with the results of most previous studies on the impacts of the COVID-19 pandemic on cancer diagnosis [4,12–14], but they are consistent with a Korean study [15]. This discrepancy among countries is presumed to be caused by differences in the magnitude of the COVID-19 pandemic and whether medical facilities were closed, and if so, for how long. In Korea, the magnitude of the COVID-19 pandemic in 2020 and 2021 was relatively small, and lockdowns of hospitals or medical facilities or restrictions on outpatient services rarely occurred [15,16]. At the end of the observation period of this study (December 31, 2021), the cumulative confirmed cases of COVID-19 per million population in Korea was 12,381, approximately 33.8% of that of the world, 6.5–7.5% of the United Kingdom and United States, 14.5% of Germany, and 90% of Japan [16]. Therefore, it is presumed that any decrease in the screening rate for CRC in Korea would not have led to an upshifting in the stage at diagnosis or treatment delays. A previous Korean study reported that no upshifting was observed among CRC patients diagnosed in 2020 [15]. However, considering that the risk of advanced CRC stage increases only when colonoscopy is delayed more than 4–9 months [10–12], this study analyzed CRC cases diagnosed up to December 2021.

The following reasons can be inferred to explain why there was no upshift of the stage at diagnosis or any treatment delay in this and previous studies in Korea. First, unlike many other countries, Korea did not implement national or regional lockdowns, except for a short-term localized lockdown in the city of Daegu for 2 weeks in May 2020 [18]. Therefore, any cancellations of cancer screening and delays in cancer diagnosis due to the closure of medical facilities or restrictions on social mobility would have had an insignificant impact on stage shifting. Second, the CRC screening rate using FIT continued to decline until 2021, but the number of diagnostic colonoscopies rebounded in 2021. Therefore, it is presumed that the early diagnosis of CRC using colonoscopy increased again within the lag time required to affect stage shifting. The Korean National Cancer Screening Survey, a nationwide, population-based cross-sectional questionnaire survey of about 4,000 respondents every year, reported that the CRC screening rate, using FIT, within the last 1 year among adults aged ≥50 years was 14.8% in 2020, the lowest rate in the past 5 years (2016–2020) [9]. Another set of data also showed that the participation rate in the Korean national CRC screening program, the NCSP, decreased from 34.8% in 2019 to 25.9% in 2020 and 27.2% in 2021. However, the number of colonoscopies performed in the study areas rebounded and increased in 2021. In Korea, the age-standardized incidence rates of CRC have also increased significantly, but since 2012, they have continued to decrease in both males and females [19]. The recent decrease in CRC incidence is presumed to be related to the increased use of colonoscopy, polyp removal, and more active treatment for precancerous lesions. The number of diagnostic and therapeutic colonoscopy procedures performed in hospitals has rapidly increased in Korea [20]. The proportion of early-stage CRC cases continues to increase, and survival has gradually improved, as is the global pattern [21]. Therefore, our findings suggest that the recent trend of decreased incidence and improved stage distribution of CRC has continued in Korea during the COVID-19 pandemic despite the decrease in the screening participation rate using FIT.

Although no evidence of delayed diagnosis and treatment for CRC was found in this study, much evidence has already been reported worldwide of the negative impacts of the pandemic on cancer diagnosis and treatment [4,13,14,22]. Because cancer patients tend to travel to other areas for the purpose of cancer treatment [23], the negative impacts of movement restrictions or social distancing during the pandemic period will be larger than for other diseases. Therefore, building infrastructure for cancer treatment in each residential area should be considered as a public health strategy to respond to future pandemics.

This study had some limitations. First, the observation period was too short to evaluate the full impacts of the pandemic, and a sufficient lag time was not considered. In particular, in Korea, it is necessary to investigate long-term effects rather than short-term effects after 1–2 years of the pandemic because the magnitude of the epidemic is relatively small, and strong social distancing interventions without a lockdown of health facilities were implemented. Therefore, although we did not find adverse impacts of the COVID-19 pandemic on CRC diagnosis and treatment, the possibility of a false negative cannot be ruled out. However, although the observation period was short to evaluate stage shifting, it was sufficient to confirm changes in the diagnostic route or treatment delay. Second, as this was a single-center study, the representativeness of the findings is weak, and the findings cannot be generalized to the Korean population. Generalization of the findings to other types of cancer should also be approached with caution. Because CRC is a relatively slowly progressing cancer, additional research is needed to evaluate faster-growing cancers, such as lung cancer.

Despite these weaknesses, this study also had several strengths. First, it was not a modeling study, but rather an observational study that analyzed real-world data on CRC. Second, various epidemiological variables such as the CRC diagnosis route, symptomatic status at the time of diagnosis, family history, and residential area were considered. Third, although it was a single-center study, it was possible to estimate the status of CRC diagnosis in the study area because it covered more than 70% of newly diagnosed CRC patients in the catchment area.

In conclusion, a negative impact of the COVID-19 pandemic on CRC diagnosis was not observed in Korea until December 2021. However, since the effect size and incubation period of effects may vary according to country or region, broader data, such as national data and longer-term epidemiological data, need to be evaluated to draw firm conclusions regarding the effect of the COVID-19 pandemic on CRC stage distribution and its clinical impact.

## Figures and Tables

**Figure f2-epih-44-e2022092:**
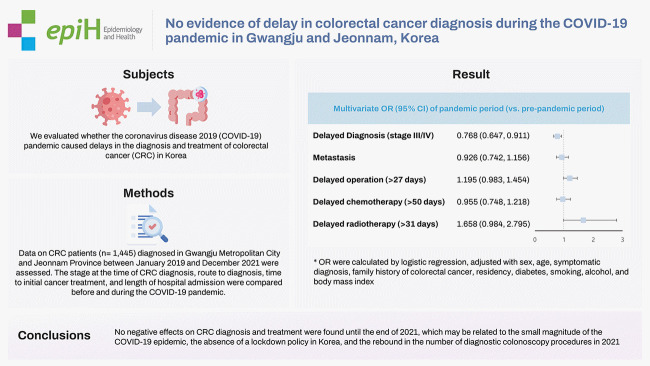


**Table 1:** Demographic and clinical characteristics according to the time of cancer diagnosis

Characteristics	Pre-pandemic period (n=934)	Pandemic period (n=1,445)	p-value
Age (yr)	68.0 [58.7–76.0]	67.0 [57.0–76.0]	0.092
Under 65	384 (41.1)	641 (44.4)	0.118
Over 65	550 (58.9)	804 (55.6)	

Sex			0.300
Male	560 (60.0)	897 (62.1)	
Female	374 (40.0)	548 (37.9)	

Residency			0.710
Metropolitan city	403 (43.1)	602 (41.7)	
City	266 (28.5)	432 (29.9)	
Rural	265 (28.4)	411 (28.4)	

Diagnosis pathway			0.791
Screening	345 (36.9)	526 (36.4)	
Non-screening	589 (63.1)	919 (63.6)	

Anatomical site			0.105
Colon	522 (55.9)	804 (55.6)	
Rectosigmoid colon	124 (13.3)	155 (10.7)	
Rectum	288 (30.8)	486 (33.6)	

Surgery			0.694
No	129 (13.8)	196 (13.6)	
Yes	805 (86.2)	1,249 (86.4)	

Chemotherapy			0.020
No	366 (39.2)	636 (44.0)	
Yes	568 (60.8)	809 (56.0)	

Radiation therapy			0.541
No	805 (86.2)	1,258 (87.1)	
Yes	129 (13.8)	18.7 (12.9)	

TNM stage			0.004
0/I/II	453 (48.7)	786 (54.7)	
III/IV	477 (51.3)	650 (45.3)	

Body mass index	23.3 [21.1–25.5]	23.3 [21.0–25.6]	0.905

Smoking			0.024
Never	573 (61.3)	819 (56.7)	
Ex/Current	361 (38.7)	626 (43.3)	

Alcohol drinking			0.921
Never	342 (36.6)	532 (36.8)	
Former/Current	592 (63.4)	913 (63.2)	

Family history of CRC			0.199
No	877 (93.9)	1,337 (92.5)	
Yes	57 (6.1)	108 (7.5)	

Diabetes mellitus			0.175
No	709 (75.9)	1,061 (73.4)	
Yes	225 (24.1)	384 (26.6)	

Length of admission (day)	15.0 [10.0–36.0]	13.0 [10.0–25.0]	0.001

Diagnosis-operation interval (day)	27.0 [17.0–40.0]	29.0 [18.0–44.0]	0.070

Diagnosis-chemotherapy interval (day)	50.0 [29.0–62.0]	49.0 [30.0–65.0]	0.557

Diagnosis-radiotherapy interval (day)	31.0 [23.0–49.0]	34.0 [27.0–50.0]	0.051

Values are presented as median [interquartile range] or number (%).

CRC, colorectal cancer.

**Table 2:** Factors associated with the risk of advanced TNM stage III/IV or metastasis

Group (vs. reference)	No. of CRC cases	Multivariate OR (95% CI)^1^	
		Stage III/IV	Metastasis
Pandemic period (vs. pre-pandemic)	1,430/928	0.768 (0.647, 0.911)	0.926 (0.742, 1.156)
Male (vs. female)	1,447/911	1.008 (0.811, 1.253)	1.244 (0.939, 1.648)
Age <65 yr (vs. ≥65 yr)	1,024/1,334	1.240 (1.042, 1.476)	1.338 (1.068, 1.675)
Symptomatic diagnosis (vs. screening)	1,490/868	2.496 (2.093, 2.978)	3.064 (2.344, 4.006)
Family history of CRC (vs. no)	165/2,193	0.825 (0.594, 1.148)	0.878 (0.567, 1.361)
Rural residency (vs. urban)	666/1,692	1.093 (0.908, 1.317)	0.924 (0.723, 1.180)
Diabetes (vs. no)	600/1,758	1.005 (0.828, 1.220)	0.941 (0.727, 1.216)
Smoker (vs. never smoker)	981/1,377	0.997 (0.802, 1.239)	0.962 (0.729, 1.270)
Alcohol drinking (vs. never drinker)	1,486/872	1.219 (1.007, 1.476)	1.169 (0.912, 1.498)
BMI <25 kg/m^2^ (vs. ≥25 kg/m^2^)	1,631/727	1.253 (1.043, 1.505)	1.541 (1.192, 1.992)

CRC, colorectal cancer; OR, odds ratio; CI, confidence interval; BMI, body mass index

1 ORs were calculated by logistic regression.

**Table 3:** Factors associated with the risk of delayed treatment for CRC

Group (vs. reference)	Multivariate OR (95% CI)^1^		
	Delayed operation (>27 day)	Delayed chemotherapy (>50 day)	Delayed radiotherapy (>31 day)
Pandemic (vs. pre-pandemic)	1.195 (0.983, 1.454)	0.955 (0.748, 1.218)	1.658 (0.984, 2.795)
Male (vs. female)	1.590 (1.300, 1.945)	0.800 (0.621, 1.030)	1.404 (0.768, 2.565)
Age <65 yr (vs. ≥65 yr)	0.887 (0.725, 1.086)	0.826 (0.645, 1.058)	0.501 (0.290, 0.865)
Symptomatic (vs. via screening)	0.811 (0.663, 0.993)	0.401 (0.310, 0.519)	0.350 (0.193, 0.635)
Family history of CRC (vs. no)	0.872 (0.603, 1.259)	1.225 (0.785, 1.913)	1.494 (0.599, 3.724)
Rural residency (vs. urban)	1.065 (0.859, 1.322)	0.976 (0.744, 1.279)	0.758 (0.439, 1.307)
Stage III/IV (vs. stage I/II)	0.999 (0.817, 1.221)	0.661 (0.510, 0.857)	1.688 (0.975, 2.901)

CRC, colorectal cancer; OR, odds ratio; CI, confidence interval.

1 ORs were calculated by logistic regression.

## References

[1] Ahmed A (2021). Corona collateral damage syndrome: perception of the damage. Indian J Crit Care Med.

[2] Wenger NS, Stanton AL, Baxter-King R, Sepucha K, Vavreck L, Naeim A (2022). The impact of COVID-19 on routine medical care and cancer screening. J Gen Intern Med.

[3] Baxter NN, Facey M, Ruco A, Baker NA, Sorvari A, Benmessaoud A (2022). Nimble Approach: fast, adapting, calculating and ethically mindful approach to managing colorectal cancer screening programmes during a pandemic. BMJ Open Gastroenterol.

[4] Harber I, Zeidan D, Aslam MN (2021). Colorectal cancer screening: impact of COVID-19 pandemic and possible consequences. Life (Basel).

[5] Cheng SY, Chen CF, He HC, Chang LC, Hsu WF, Wu MS (2021). Impact of COVID-19 pandemic on fecal immunochemical test screening uptake and compliance to diagnostic colonoscopy. J Gastroenterol Hepatol.

[6] Vives N, Binefa G, Vidal C, Milà N, Muñoz R, Guardiola V (2022). Short-term impact of the COVID-19 pandemic on a population-based screening program for colorectal cancer in Catalonia (Spain). Prev Med.

[7] Lantinga MA, Theunissen F, Ter Borg PC, Bruno MJ, Ouwendijk RJ, Siersema PD (2021). Impact of the COVID-19 pandemic on gastrointestinal endoscopy in the Netherlands: analysis of a prospective endoscopy database. Endoscopy.

[8] Chen SL, Yan BM, Infantolino A, Tofani CJ (2022). Public interest in colonoscopy and colon cancer decreased following the onset of the COVID-19 pandemic. Dis Colon Rectum.

[9] Lee K, Lee YY, Suh M, Jun JK, Park B, Kim Y (2022). Impact of COVID-19 on cancer screening in South Korea. Sci Rep.

[10] Forbes N, Hilsden RJ, Martel M, Ruan Y, Dube C, Rostom A (2021). Association between time to colonoscopy after positive fecal testing and colorectal cancer outcomes: a systematic review. Clin Gastroenterol Hepatol.

[11] Lee YC, Fann JC, Chiang TH, Chuang SL, Chen SL, Chiu HM (2019). Time to colonoscopy and risk of colorectal cancer in patients with positive results from fecal immunochemical tests. Clin Gastroenterol Hepatol.

[12] Ricciardiello L, Ferrari C, Cameletti M, Gaianill F, Buttitta F, Bazzoli F (2021). Impact of SARS-CoV-2 pandemic on colorectal cancer screening delay: effect on stage shift and increased mortality. Clin Gastroenterol Hepatol.

[13] Maringe C, Spicer J, Morris M, Purushotham A, Nolte E, Sullivan R (2020). The impact of the COVID-19 pandemic on cancer deaths due to delays in diagnosis in England, UK: a national, population-based, modelling study. Lancet Oncol.

[14] Losurdo P, Samardzic N, Di Lenarda F, de Manzini N, Giudici F, Bortul M (2022). The real-word impact of breast and colorectal cancer surgery during the SARS-CoV-2 pandemic. Updates Surg.

[15] Lim JH, Lee WY, Yun SH, Kim HC, Cho YB, Huh JW (2021). Has the COVID-19 pandemic caused upshifting in colorectal cancer stage?. Ann Coloproctol.

[16] Our World in Data Cumulative confirmed COVID-19 cases per million people.

[17] Gwangju-Jeonnam Cancer Registry (2022). Annual report of cancer incidence in Gwangju and Jeonnam 2019.

[18] Dighe A, Cattarino L, Cuomo-Dannenburg G, Skarp J, Imai N, Bhatia S (2020). Response to COVID-19 in South Korea and implications for lifting stringent interventions. BMC Med.

[19] Kweon SS (2018). Updates on cancer epidemiology in Korea, 2018. Chonnam Med J.

[20] Jung Y, Kim JW, Im JP, Cho YK, Lee TH, Jang JY (2022). Safety of gastrointestinal endoscopy in Korea: a nationwide survey and population-based study. J Korean Med Sci.

[21] Jiang Y, Yuan H, Li Z, Ji X, Shen Q, Tuo J (2021). Global pattern and trends of colorectal cancer survival: a systematic review of population-based registration data. Cancer Biol Med.

[22] Mazidimoradi A, Tiznobaik A, Salehiniya H (2022). Impact of the COVID-19 pandemic on colorectal cancer screening: a systematic review. J Gastrointest Cancer.

[23] Kim W, Han KT, Kim S (2021). Do patients residing in provincial areas transport and spend more on cancer treatment in Korea?. Int J Environ Res Public Health.

